# Rs46522 in the Ubiquitin-Conjugating Enzyme E2Z Gene Is Associated with the Risk of Coronary Artery Disease in Individuals of Chinese Han Population with Type 2 Diabetes

**DOI:** 10.1155/2017/4501794

**Published:** 2017-08-03

**Authors:** Difei Lu, Jia Huang, Xiaowei Ma, Nan Gu, Junqing Zhang, Hong Zhang, Xiaohui Guo

**Affiliations:** Endocrinology Department, Peking University First Hospital, No. 8, Xishiku Street, Beijing 100034, China

## Abstract

**Aims:**

We investigated the association between ubiquitin-conjugating enzyme E2Z (UBE2Z) gene SNP rs46522 and the risk of CAD in a Chinese Han population with type 2 diabetes and explored a possible interactive effect with environmental risk factors of CAD.

**Methods:**

665 patients with T2D were enrolled; 390 were CAD patients and 275 were non-CAD patients. Genotype analysis of rs46522 (T>C) was performed using PCR-RFLP.

**Results:**

The SNP rs47522 was associated with the risk of CAD supposing recessive inheritance model (TT versus CC+CT, OR′ = 1.277, 95%CI′ 1.039–1.570, *p*′ = 0.020) and codominant model (TT versus CT, OR′ = 1.673, 95%CI′ 1.088–2.570, *p*′ = 0.019) after adjustment for confounders of CAD. A synergistic effect of rs46522 and BMI was discovered (*β* = 0.012, *p for interreaction* = 0.028). In subgroup analysis, minor allele T was significantly associated with CAD in overweight and obesity subgroup (*p* = 0.034), and the association was also proved in recessive model (OR = 1.537, 95%CI 1.075–2.196, *p* = 0.018). Smokers with genotype TT had threefold risk of CAD in comparison to nonsmokers with genotype TC or CC (*p* < 0.001).

**Conclusions:**

The SNP rs46522 in UBE2Z gene is associated with the risk of CAD in the individuals of Chinese Han descent with type 2 diabetes and is of synergistic effect with BMI.

## 1. Introduction

Diabetes mellitus is one of the most harmful metabolic diseases worldwide, and a major challenge to public health, especially in China [1]. Coronary artery disease (CAD), as a main complication and comorbidity of T2D, is a leading cause of mortality. Both environmental and genetic factors play a critical role in the development of CAD [2]. It is estimated that heritable factors account for 30–60% of the interindividual variation in the pathogenesis of coronary heart disease [3]. To date, genome-wide associated studies (GWAS) have identified more than 60 CAD risk loci, and a series of following studies proved their susceptibility to CAD in different ethnics [4]. In a recent large-scale association analysis, rs46522 on chromosome 17q21.32, in the intronic region of ubiquitin-conjugating enzyme E2Z (UBE2Z) encoding gene, was associated with risk of CAD in European descent [5]. Meanwhile, the susceptibility is also proved in an Iranian population [6]. However, a contradicted conclusion was conducted in a Japanese population, indicating the association of rs46522 and CAD might be ethnic-specific [7]. UBE2Z is physically located near GIP gene along chromosome 17, while it is known that GIP plays a protective role in atherosclerosis and obesity. In this study, our aim was to evaluate whether or not rs46522 close to GIP was associated with CAD risk in the Chinese subjects with type 2 diabetes.

## 2. Materials and Methods

### 2.1. Ethics Statement

The study was designed in accordance with the Helsinki Declaration. The research protocol and informed consent forms were approved by the research ethics committee of Peking University First Hospital. Before recruited, all subjects gave informed written consent form.

### 2.2. Patient Selection

Diagnosis of type 2 diabetes was based on the 1999 WHO diagnostic criteria, which is defined as fasting blood glucose (FBG) of 7.0 mmol/L (126 mg/dL) or over and/or 2 hours postprandial blood glucose (PBG) of 11.1 mmol/L (200 mg/dL)) or over. Subjects were selected from those patients who underwent coronary angiography or coronary artery CT scan during their hospitalization in Peking University First Hospital. 665 unrelated Chinese Han individuals with T2D were enrolled, 390 of whom were CAD-positive and 275 were CAD-negative. Subjects with CAD were defined as a stenosis size of 50% or above in at least one of major coronary arteries or their main branches on cardioangiography at Peking University First Hospital. Non-CAD subjects were confirmed with cardioangiography or coronary artery CT scan with the stenosis less than 50%. Once recruited, complete medical records written by residents of these patients were collected including the following data: age, gender, ethnicity, family, social and medication history, vital signs, body weight, body height, and smoking status (“ever”or “never,” “ever”defined as having smoked more than 1 cigarette per day for more than 1 year).

### 2.3. Single Nucleotide Polymorphism Genotyping

Peripheral blood was withdrawn and stored in EDTA-containing tubes. Genomic DNA was isolated with the salting-out method from peripheral blood leukocytes [8]. The extracted DNA was stored at −20°C until analyzed.

The SNP rs46522 (T>C) at an intron of UBE2Z gene is located on chromosome 17q21.32. It was genotyped using polymerase chain reaction following restriction fragment length polymorphism (PCR-RFLP). Primers were designed as 5′-CATAGTGAGGTGGAGCAGCA and 5′-ACGCAACCAGTAAGGACAGG using Primer 3.0 software. A 25 *μ*L PCR system consisted of 1 *μ*L DNA template of each subject, 1 *μ*L primers of both forward and reverse ones (5 pmol/*μ*L), 12.5 *μ*L 2 × PCR Mix of Taq polymerase and dNTP, and 10.5 *μ*L double distilled water. Annealing temperature was set at 61°C for 35 cycles. The reaction system of RFLP was constituted of 7.5 *μ*L PCR product, 0.5 *μ*L restriction endonuclease (FokI, NEB corporation), 2 *μ*L 10× buffer (NEB corporation), and 10 *μ*L double distilled water. After 2 hours of water bath heating (65°C), RFLP product was analyzed using agarose gel electrophoresis. Furthermore, direct sequencing method was performed in 5% of randomized selected samples in order to minimize bias for the quality control.

### 2.4. Statistical Analysis

The statistical analyses were carried out using the Statistical Package for the Social Sciences for Windows (SPSS), version 16.0 (IBM, Chicago, IL, USA). Statistical significance was defined as *p* value of <0.05, and odds ratios (ORs) were calculated with 95% confidence intervals (CIs). Continuous variables were expressed as mean ± SD (standard deviation) and were analyzed with Student's *t*-test or one-way ANOVA. Categorical variables were presented as percentage of cases and were analyzed with the Pearson chi-square test. Genotype distribution of the SNP rs46522 was tested for Hardy-Weinberg equilibrium. The associations between genotypes and CAD were analyzed using multiple logistic regression analysis and were further adjusted for potential confounders including age, gender, history of hypertension and diabetes, lipid levels, fasting blood glucose, BMI, and smoking status.

## 3. Results

### 3.1. Characteristics of the Study Population

A total of 665 patients with T2D were recruited in this study. The demographic data and clinical characteristics were not significantly different between the CAD and non-CAD groups but in gender, age, and HbA1c, lipid profile, and smoking status (Table 1). However, the values of HbA1c and lipid profile were shown after intervention.

### 3.2. Genotyping Results and Association Analyses

The observed genotype frequency of rs46522 in non-CAD group was consistent with the Hardy-Weinberg equilibrium. The rate of genotyping success was greater than 98% for the SNP rs46522. The genotypes were consistent with the results from direct sequencing for the randomly chosen samples, and the consistent rate was 95%. There were no significant differences between CAD and non-CAD groups in the distribution of allele frequencies and genotype frequencies.

Assumed in recessive inheritance model, genotype TT was associated with a significantly higher risk of CAD compared to genotypes TC and CC (*p* = 0.044), and the significance remained after adjustment for the confounders including age, gender, history of hypertension and diabetes, smoking status, BMI, fasting glucose, and lipid levels (*p* = 0.020, Table 2). Power was calculated as 80.7%, indicating that the possibility of type II error was less than 20%.

In codominant inheritance model, the carriers of genotype TT had a higher risk of CAD compared to those of genotype TC after adjustment for the above covariates (*p*′ = 0.019, Table 3). No significant difference was found in the two groups in a dominant inheritance model.

### 3.3. Subgroup Analysis

Both genetic variations and environmental factors contributed to the pathogenesis of CAD. While taken as a successive covariate, BMI exhibited a synergistic effect with rs46522 (*β* = 0.012, *p for interreaction* = 0.028) on the CAD risk.

The subjects were further stratified according to the environmental risk factors, BMI and smoking habits for subgroup analyses. In overweight and obesity subgroup, defined as BMI of 24 kg/m^2^ or above, the frequency of allele T demonstrated a higher risk of CAD than allele C (OR = 1.341, 95%CI 1.023–1.757, *p* = 0.034). Also, the carriers of genotype TT had a higher risk of CAD than carriers of genotypes TC and CC in overweight and obesity subgroup, assumed in recessive model (OR = 1.537, 95%CI 1.075–2.196, *p* = 0.018, Table 4).

Smoking is a conventional risk factor of CAD. The smokers with genotype TT had a risk of CAD even twofold higher compared to nonsmokers with genotypes TC and CC (OR = 3.001, 95%CI 1.883–4.785, *p* < 0.001, Table 5). However, this significance no longer remained after being adjusted for the conventional confounding factors for CAD.

## 4. Discussion

Genetic variants including SNPs are involved in multiple aspects of the pathogenesis of human diseases. In recent years, the functional research of SNPs has increased rapidly, but the genetic background for certain diseases is even more beyond our knowledge and yet to be explained.

So far, researches on genetic factors of CAD have been performed in general populations and revealed new loci associated with CAD. Meanwhile, a few convincing studies suggested that some genes affect both atherosclerosis and diabetes [9]. Interestingly, a number of the studies including some of ours have shown that CAD risk may be partially different in diabetic individuals than in general population in terms of genetic susceptibility [10].

UBZ2E functions as a ubiquitin-conjugating enzyme to activate ubiquitin-like modifier (UBL), which is transferred onto the target protein with an isopeptide bond formation [11]. As of date, GWAS discovered that rs46522 in UBE2Z gene contributed to CAD susceptibility mainly in Caucasians [5]. Several population-based studies demonstrated that the associations between rs46522 and CAD varied across different descents [6, 7]. In the study, SNP rs46522 in UBE2Z was found contributing to the development of CAD in Chinese Han population with type 2 diabetes. Carriers of genotype TT had significantly higher risk of CAD in comparison with those of genotypes TC and CC, before and even after adjustment for conventional confounders of CAD. Our results are consistent with the observations in Europeans and a case-control study in Iranian population [5, 6]. However, a conflicted conclusion arose from a cohort study in general Japanese population [7]. We postulated that the different results occurred primarily due to varied populations. The study subjects in our research were type 2 diabetes patients of Chinese Hans, while the Japanese study mainly focused on the elderly of Japan.

The potential mechanism by which the genetic variations in UBE2Z gene contribute to CAD risk was not yet clearly explicated. As the SNP rs46522 is located in an intronic region of UBE2Z gene, one possible explanation is that rs46522 is in strong linkage disequilibrium with the causal SNPs of gastric inhibitory polypeptide (GIP) gene, while the encoding protein GIP could modulate glucose and lipid metabolism. GIP gene is located on chromosome 17q21.32 and contains 6 exons. It was proved that functional variant SNPs in GIP gene were associated with cardiovascular disease in a small-scale case-control study [12]. Interestingly, the leading CAD-associated SNPs in GIP gene from previous studies were found to be in high linkage disequilibrium with 2 functional variants in GIP gene: Ser103Gly (rs2291725) and a slice site variant (rs2291726) in a recent study [13]. Further study on GIP gene variants and CAD risk could be conducted in Chinese population with or without type 2 diabetes to prove the hypothesis.

Meanwhile, efforts were made to explore if there was a possible association between GIP gene variants and the risk of type 2 diabetes. In a case-control analysis in a South Indian population, no significant association was found between common variants in GIP (rs2291725 and rs2291726) and diabetes [14]. If the association of GIP gene and CAD was proved, a tentative inference on this conclusion is that the mechanism of GIP gene influencing the risk of CAD might be independent of the development of diabetes.

As a coronary artery disease-related variant, rs46522 at the UBE2Z locus yielded significant association with type 2 diabetes in a genome-wide association study [15]. Exploration could be made to discover the mechanism of UBE2Z locus affecting both CAD and type 2 diabetes in a molecular level.

The potential interactive effect of rs46522 was investigated with traditional environmental confounders in CAD risk. BMI, as one of the known CAD risk factors, displayed a possible effect of interaction with the SNP rs46522 in the study (*β* = 0.012, *p for interreaction* = 0.028). In other words, every 1 kg/m^2^ increase in BMI resulted in additional 1.2% higher CAD risk in the given genetic group. To date, accumulating evidence indicated that BMI and waist circumference were significantly related to insulin resistance and chronic inflammatory state [16], which might lead to cardiovascular diseases in conjunction with genetic factors.

As a main risk factor of CAD, smoking was also taken into consideration in the study. The study showed that the smokers with genotype TT at rs46522 had significant twofold increase in CAD risk compared to nonsmokers with genotype TC/CC, although the difference did not exist anymore after adjustment.

In general, the study can be interpreted that rs46522 is associated with the CAD risk in patients with type 2 diabetes, and increased BMI and smoking could increase CAD risk, especially in the individuals with higher genetic susceptibility. Clinically, the study provides strong evidence for diabetes education that body weight management and smoking cessation can significantly benefit the patients from CAD.

However, the following limitations should be acknowledged in the study. The number of enrolled individuals was relatively small, especially the sample size of non-CAD group. Also, clinical values of CAD and non-CAD groups were not perfectly matched, which might bias the results even though adjustments were performed. Functional study and perspective study should further be conducted on the genetic variants in UBE2Z and GIP gene to discover the underlying pathway and confirm the predicting value for CAD in Chinese population, respectively.

In conclusion, the study showed that rs46522 in UBE2Z gene contributed to the development of CAD in individuals of Chinese Han descent with type 2 diabetes and had an interactive effect with BMI on CAD risk. Genotype TT at rs46522 is a risk factor for CAD, especially in patients with smoking habits.

## Figures and Tables

**Table 1:** Clinical characteristics of CAD group and non-CAD group.

	CAD group	Non-CAD group	
	*n* = 390	*n* = 275	*p* value
Gender (M/F)	257/133	118/158	<0.001
Age (yr)	64 (57–72)	61 (55–69)	0.001
BMI (kg/m^2^)	26.39 ± 3.37	26.19 ± 3.61	0.472
History of T2D (yr)	8 (3–13)	7 (3–12)	0.769
History of hypertension (%)	76.2	70.7	0.110
Fasting plasma glucose (mmol/L)	6.45 (5.59–7.80)	6.42 (5.47–7.64)	0.263
HbA1c (%)	6.8 (6.3–7.8)	6.7 (6.1–7.5)	0.080
Triglyceride (mmol/L)	1.39 (0.96–1.93)	1.40 (0.97–2.03)	0.615
Total cholesterol (mmol/L)	4.00 ± 1.04	4.24 ± 0.92	0.004
LDL-C (mmol/L)	2.26 (1.79–2.91)	2.45 (1.91–2.94)	0.071
HDL-C (mmol/L)	0.95 (0.80–1.10)	1.03 (0.86–1.23)	<0.001
Smoking status (%)	51.5	34.1	<0.001

BMI: body mass index; FPG: fasting plasma glucose; CAD: coronary artery disease; T2D: type 2 diabetes; HDL-C: high-density lipoprotein cholesterol; LDL-C: low-density lipoprotein cholesterol. The fasting plasma glucose values, lipid profile, and HbA1c were after intervention.

**Table 2:** Genotype distribution of rs46522 between CAD and non-CAD groups in recessive inheritance model.

Genotype frequency	CAD group *n* (%)	Non-CAD group *n* (%)	OR (95%CI)	*p* value	OR′ (95%CI′)	*p*′ value
T/T	194 (49.7)	115 (41.8)				
C/T+C/C	196 (50.3)	160 (58.2)	1.377 (1.009–1.880)	**0.044**	1.277 (1.039–1.570)	**0.020**

CAD: coronary artery disease. OR′, 95%CI′, and *p*′ were after adjustment with age, gender, history of hypertension and diabetes, smoking status, BMI, fasting glucose, and lipid levels.

**Table 3:** Genotype distribution of rs46522 between CAD and non-CAD groups in codominant inheritance model.

Genotype frequency	CAD group *n* (%)	Non-CAD group *n* (%)	OR (95%CI)	*p* value	OR′ (95%CI′)	*p*′ value
T/T	194 (49.7)	115 (41.8)				
C/T	165 (42.3)	135 (49.1)	1.380 (0.998–1.908)	0.051	1.673 (1.088–2.570)	**0.019**
C/C	31 (7.9)	25 (9.1)	1.360 (0.765–2.418)	0.293	1.182 (0.799–1.749)	0.402

CAD: coronary artery disease. OR′, 95%CI′, and *p*′ were after adjustment with age, gender, history of hypertension and diabetes, smoking status, BMI, fasting glucose, and lipid levels.

**Table 4:** Genotype distribution of rs46522 between CAD and non-CAD groups in recessive model in overweight and obesity subgroup.

Genotype frequency	CAD group *n* (%)	Non-CAD group *n* (%)	OR (95%CI)	*p* value	OR′ (95%CI′)	*p*′ value
T/T	156 (52.0)	86 (41.3)	1.537 (1.075–2.196)	**0.018**	1.202 (0.950–1.521)	0.125
C/T+C/C	144 (48.0)	122 (58.7)				

CAD: coronary artery disease. OR′, 95%CI′, and *p*′ were after adjustment with age, gender, history of hypertension and diabetes, smoking status, fasting glucose, and lipid levels.

**Table 5:** The subgroup analysis in smoking status and genotypes of rs46522.

	CAD group *n* (%)	Non-CAD group *n* (%)	OR (95%CI)	*p* value	OR′ (95%CI′)	*p*′ value
Nonsmoking + T/C or C/C	102 (26.2)	103 (37.5)	1	1	1	1
Nonsmoking + T/T	87 (22.3)	79 (28.7)	0.899 (0.597–1.354)	0.611	0.969 (0.747–1.257)	0.812
Smoking + T/C or C/C	94 (24.1)	57 (20.7)	1.665 (1.085–2.555)	**0.019**	1.076 (0.746–1.552)	0.693
Smoking + T/T	107 (27.4)	36 (13.1)	3.001 (1.883–4.785)	**<0.001**	1.330 (0.884–2.001)	0.171

CAD: coronary artery disease. OR′, 95%CI′, and *p*′ were after adjustment with age, gender, history of hypertension and diabetes, BMI, fasting glucose, and lipid levels.
